# Nanocomposite Hydrogels and Extracellular Matrix—Advantages and Associated Risks

**DOI:** 10.3390/gels9090754

**Published:** 2023-09-16

**Authors:** Garry Kerch

**Affiliations:** Faculty of Materials Science and Applied Chemistry, Riga Technical University, P. Valdena 3, 1048 Riga, Latvia; garrykerch@inbox.lv

**Keywords:** extracellular matrix, stiffness, cancers, nanocomposite hydrogels, internal stresses, state of water, mechanotransduction

## Abstract

Hydrogels can be considered as mimics of the extracellular matrix (ECM). Through integrins, the cytoskeleton is connected to the ECM, and cytoskeleton tension depends on ECM stiffness. A number of age-related diseases depend on cellular processes related to cytoskeleton function. Some examples of cancer initiation and progression and heart disease in relation to ECM stiffness have been analyzed. The incorporation of rigid particles into the ECM can increase ECM stiffness and promote the formation of internal residual stresses. Water migration, changes in water binding energy to biomactomolecules, and changes in the state of water from tightly bound water to free and loosely bound water lead to changes in the stiffness of the ECM. Cardiac tissue engineering, ECM stiffness and cancer, the equivalence of ECM stiffness, oxidative stress, inflammation, multi-layer polyelectrolyte complex hydrogels and bioprinting, residual internal stresses, viscoelastic hydrogels, hydrogel nanocomposites, and the effect of water have been reported. Special attention has been paid to the role of bound water and internal stresses in ECM stiffness. The risks related to rigid particle incorporation into the ECM have been discussed. The potential effect of polyphenols, chitosan, and chitosan oligosaccharide on ECM stiffness and the potential for anti-TNF-α and anti-NF-κB therapies have been discussed.

## 1. Introduction

Hydrogels can be used as artificial extracellular matrices in tissue engineering in the development of scaffolds for biomimetic three-dimensional cell culture. The importance of and challenges in the design and development of hydrogels that mimic the mechanical and biological properties of the native extracellular matrix have been reviewed [1,2].

The design of hydrogels with tunable physiochemical and biological properties and their potential applications in regenerative medicine were discussed by researchers from Harvard University and Massachusetts Institute of Technology in 2014 [3]. Hydrogels resemble the native extracellular matrix, and their use as “three-dimensional (3D) matrices for tissue engineering, drug-delivery vehicles, composite biomaterials, and as injectable fillers in minimally invasive surgeries” was characterized as promising for future applications. All the above-mentioned potential applications contain rigid fillers that can increase the extracellular matrix stiffness and therefore the potential advantages and risks have to be estimated.

The function and properties of hydrogels containing matrix-like molecules, hydrogels containing a decellularized matrix, hydrogels derived from the decellularized matrix, and decellularized tissues as reimplantable matrix hydrogels have also been discussed [4]. 

Poor mechanical strength is mentioned as one of the major limitations in the application of tissue engineering [5]. The attempts to improve the mechanical properties can lead to the risk of age-related disease initiation as a result of tissue stiffening. The aim is to attract attention to the as yet unpublished potential risks of extracellular matrix stiffening by entrapped rigid particles, leading to the initiation and accelerated progression of cancers, cardiovascular disease, and some other age-related diseases. The risks of the initiation and progression of severe age-related diseases associated with the incorporation of rigid particles into the ECM and the related increase in elasticity modulus may be suggested for consideration.

## 2. Results and Discussion

### 2.1. Cardiac Tissue Engineering

The application of hydrogels in the repair and regeneration of damaged hearts was discussed [6,7,8].

Researchers at the University of Colorado Boulder discussed the three-dimensional encapsulation of adult mouse cardiomyocytes in hydrogels with tunable stiffness [6]. The morphology and the function of the cardiomyocytes depended on the mechanical properties of their microenvironment. The authors used photo-clickable thiol-ene poly (ethylene glycol) (PEG) hydrogels for the three-dimensional cell culture of adult mouse cardiomyocytes. The stiffness of the PEG hydrogels could be tuned to mimic the microenvironments of cells, and cell function was dependent on the PEG hydrogel stiffness.

Injectable conductive carbon and metal-based nanocomposite hydrogels for cardiac tissue engineering were reviewed [8]. It would be beneficial in the near future to study the role and impact of nanoparticle rigidity quantitatively and in more detail, especially in the context of mechanical moduli improvement and in cardiac tissue engineering.

UV-crosslinkable gold nanorod (GNR)-incorporated gelatin methacrylate (GelMA) hybrid conductive hydrogels were investigated by researchers at Arizona State University for engineering cardiac tissue constructs [9]. Cardiac patches with superior electrical and mechanical properties were suggested to be developed using nanoengineered GelMA-GNR hybrid hydrogels.

The applications of photopolymerized hydrogels in tissue engineering were also reviewed [10].

Although it was already reported in 1997 that fibroblasts and epithelial cells on flexible substrates showed reduced spreading compared with cells on rigid substrates [11], one recently published review [12] stated that cell–matrix interaction “remains in its infancy, and the detailed molecular mechanisms are still elusive regarding scaffold-modulated tissue regeneration”. Injectable biodegradable hydrogels composed of gelatin–hydroxyphenylpropionic acid conjugate with tunable mechanical stiffness for the stimulation of the neurogenesis differentiation of human mesenchymal stem cells in a 3D culture were described, and it was observed that the rate of human mesenchymal stem cell proliferation increased with decreasing the hydrogel stiffness. The modulation of mesenchymal stem cell chondrogenesis in a tunable hyaluronic acid hydrogel microenvironment was reported [13]. Silk hydrogels with variable stiffness and growth factors were reported in one study of human mesenchymal stem cell cultures [14].

At first, it was discovered that the cellular processes depend on the substrate stiffness. However, it was also concluded that cell mechanosensing is regulated by substrate strain energy rather than stiffness [15]. It was demonstrated that hydrogels can properly mimic the extracellular matrix (ECM) [16].

### 2.2. Multi-Layer Polyelectrolyte Complex Hydrogels and Bioprinting

Multi-layered hydrogels prepared using layer-by-layer self-assembly can be used for biomedical applications [17].

Layer-by-layer-assembled drug delivery systems can be developed and applied for cancer treatment and in cardiovascular surgery for the surface functionalization of various implants. LbL assembly of soft polymers on nanoparticles can decrease the risks of adverse effects of rigid particles on biological processes caused by high stiffness. The advantage of LbL assembly technology is that it allows controlling the thickness and surface charge of oppositely charged polyelectrolytes near the rigid surface by tuning the assembling conditions. Electrostatic force, hydrogen bonding, charge transfer interaction, and covalent bonding play a role in LbL assembly. Various synthetic, such as doxorubicin or paclitaxel, and natural, such as curcumin, drugs for cancer treatment and their combinations can be loaded into different layers of polyelectrolyte multilayers.

At present, most investigations of LbL-assembled drug delivery systems are still in the pre-clinical stage.

Polyelectrolyte multilayers with tunable hydration and stiffness also have potential to be used in drug-eluting stents as tailored blood-contacting coatings and in novel atraumatic surgical instruments. The prevention of in-stent restenosis and thrombosis also has potential to be achieved by using polyelectrolyte multilayers.

The 3D printing, design, and emerging biomedical applications of hydrogels were presented in a very detailed review [18]. Laser printing (stereolithography, two-photon polymerization), extrusion printing (3D plotting, direct ink writing), inkjet printing, 3D bioprinting, 4D printing, and 4D bioprinting were mentioned in this review. Hydrogel-forming polymers that were described include biopolymers, synthetic polymers, polymer blends, nanocomposites, functional polymers, and cell-laden systems. Biomedical applications in tissue engineering, regenerative medicine, cancer research, in vitro disease modeling, high-throughput drug screening, and surgical preparation were discussed.

The three-dimensional (3D) printing of hydrogel composite systems such as particle-reinforced hydrogel composites, fiber-reinforced hydrogel composites, and anisotropic filler-reinforced hydrogel composites were reviewed [19]. It would be beneficial to explain the role of fillers in the above-mentioned hydrogel composites in more detail. 

### 2.3. Residual Internal Stresses

The residual tensile and compressive stresses arise near filler particles in polymer composites [20]. Due to mechanotransduction, such mechanical stresses arise similarly in the ECM and can be transferred into cytoskeleton tension with the activation of the NF-κB signaling pathway and changing gene expression. The presence of soft components at small concentrations in the rigid matrix results in the development of compressive stress due to differences in the thermal expansion coefficients and leads to maximum dependence of the elasticity modulus on the filler concentration. The temperature changes result in changes of the residual stresses values and reinforcement values. The migration of low-molecular-weight molecules with higher thermal expansion coefficients to interfaces can increase ECM stiffness due to the development of compressive residual stresses.

The stresses and properties of a composite gel consisting of a poly(vinyl alcohol (PVA) matrix filled with poly(acrylic acid) (PAA) microgel particles were reported [21]. The swelling of the PAA particles was limited by the tensile stress developing in the PVA matrix. The maximum tensile stress was found to increase with the stiffness of the PVA matrix and to decrease with increasing crosslink density of the PAA.

It can be suggested that stresses in the ECM due to mechanotransduction can be translocated to cells and cause changes in important cellular processes.

Cells respond to mechanical microenvironment cues via Rho signaling [22]. Rho GTPase is closely related to tumorigenesis, invasion, and metastasis. The Rho subfamily is involved in the formation of tensile fibers and focal adhesion complexes (FACs). The Rho signaling pathway plays an important role in the development of cancer, and it is also expected to be possible treat cancer by developing inhibitors of the Rho signaling pathway [23]. 

### 2.4. Viscoelastic Hydrogels

A group of authors from Stanford University, Harvard University, The University of Queensland, and the University of Pennsylvania discussed the effect of ECM viscoelasticity in hydrogels on cellular behavior [24]. 

Viscoelastic hydrogels with tunable stress relaxation were developed, and the stress relaxation was shown to regulate cell differentiation, spreading, and proliferation [25]. The faster relaxation in alginate-PEG hydrogels was associated with increased spreading and proliferation of fibroblasts and enhanced osteogenic differentiation of mesenchymal stem cells (MSCs). It can be considered that stress relaxation is related to residual stresses [20].

It has been reported that stress relaxation of semi-flexible chain segments in hydrogels made from the polyelectrolyte complexation of sodium hyaluronate (HA) and chitosan *increases* with increasing temperature and salt concentration [26]. 

The stiffness of multilayer stent nanocoatings depends on their hydration degree and plays an important role in the prevention of protein adsorption and platelet adhesion [27].

### 2.5. Hydrogel Nanocomposites 

It has been reported in an article published by researchers from the University of Cambridge that hydrogels resemble the ECM and can support cell proliferation when they are used as tissue engineering scaffolds and that nanofibers improve both the mechanical properties and biofunctionality of hydrogels [28]. 

However, it still must be taken into consideration that nanofibers localized in the ECM can increase viscosity and stiffness due to the presence of rigid surfaces at interfaces of the heterogeneous matrix and initiate harmful processes in cells related to the initiation of severe age-related diseases through mechanotransduction. Therefore, the functionality improvement must be confirmed in additional studies. 

For example, recently, a novel organic–mineral nanofiber/hydrogel of chitosan-polyethylene oxide (CS-PEO)/nanoclay–alginate (NC-ALG) was prepared, and the effects of nanoclay particles on the mineralization and biocompatibility of the scaffold were investigated. The authors concluded that the CS-PEO/ALG scaffold is suitable for bone tissue regeneration because it enhances bone-like apatite formation [29]. The potential influence of nanoclay particles on the signaling pathways and gene expression due to stiffness increase has not been discussed in detail, though it can be expected that particles incorporated into the ECM play an essential role in the regulation of cellular processes, especially in the soft tissues.

The interaction between the ECM and nanoparticles was discussed [30]. It was reported that the risk of toxic effects of nanoparticles increases with decreasing particle size. Nanoparticle-dependent signaling can alter the ratio between ECM-degrading enzymes called matrix metalloproteinases (MMPs) and tissue inhibitors of matrix proteases (TIMPs), resulting in the facilitation of inflammatory processes and ECM pathological changes. It must be taken into consideration that ECM stiffness can modulate the expression and secretion of MMPs and TIMPs. It has been concluded that due to “continuously growing release of various nanoparticles into the environment there is a demanding necessity to define and categorize ECM/nanoparticles interactions and to examine their relevance regarding toxicity and inflammation in various biological processes”.

The extracellular matrix stiffness regulates the induction of malignant phenotypes in the mammary epithelium. Researchers at Stanford University confirmed that cells sense the stiffness of their surrounding ECM through Rho-mediated contraction of the actin-myosin cytoskeleton [31].

A stiffness-tunable graphene oxide/polyacrylamide composite scaffold was fabricated to investigate the effect of substrate stiffness on cytoskeleton assembly and specific gene expression during cell growth by researchers at Tsinghua University, China [32]. Cells sense the ECM and translate mechanical stresses into biochemical signals, activating diverse signaling pathways or changing the intracellular calcium concentration. Cellular functions such as migration, proliferation, differentiation, and apoptosis are modulated by mechanotransduction. It was suggested that defects in mechanotransduction are able to cause diverse diseases including cancer progression and metastasis [33]. Not only mechanotransduction defects but a number of other causes such as increased tissue stiffness, increased crosslinking by glycation end products, increased collagen content, dehydration, and release of bound water molecules can initiate cancer and a number of age-related diseases.

### 2.6. Effect of Water

The dehydration, glycation, crosslinking, and redistribution of bound water molecules and entropy-driven release of tightly bound water molecules are the processes leading to ECM stiffening [27,34,35,36,37,38]. 

Enhanced mobility of intracellular water for cancer cells vs. non-malignant cells investigated using quasi-elastic neutron scattering (QENS) was reported [39]. The binding energy of water molecules to biomacromolecules decreases in cancer cells, and it is possible to suggest that a decrease in binding energy results in the transformation of a healthy cell into a cancer cell. The role of water binding energy in the initiation of various diseases was reviewed [35,36,37].

Water response to the anticancer drug cisplatin was investigated using neutron scattering spectroscopy performed at the ISIS Pulsed Neutron and Muon Source of the Rutherford Appleton Laboratory in a low-energy OSIRIS high-flux indirect-geometry time-of-flight spectrometer, and intracellular water mobility reduction was observed [40]. Therefore, it can be suggested that the application of cisplatin increases water binding energy.

It has been concluded that normal-to-malignant transformation is a poorly understood process associated with and dependent on the dynamical behavior of water, so two different water populations were considered: “one displaying bulk-like dynamics (extracellular and intracellular/cytoplasmic) and another with constrained flexibility (extracellular/interstitial and intracellular/hydration layers)” [41].

It was shown in vivo that in low magnetic fields, tumor cells display much higher proton relaxation time T_1_ values compared with the values shown by healthy cells, and it was found that the elongation of the relaxation time T_1_ can be associated with the tumor aggressiveness [42]. The elongation of T_1_ can be related to transformation of tightly bound water into slightly bound water. Therefore, it can be suggested that the release of tightly bound water can increase the risk of cancer initiation. The bound water content in the ECM can be related to cell adhesion, and the decrease in cell adhesion can lead to an increase in cells migration and cancer metastasis.

It was shown that not only bulk but also nanoscale properties of collagen fibrils play a significant role in determining cell phenotype. The collagen fibrils can be dehydrated, and smooth muscle cells spread and proliferate extensively when seeded on these dehydrated fibrils, which are mechanically stiffer compared to fully hydrated fibrils. The authors suggested that the nanoscale rigidity of collagen fibrils can cause these cells to assume a proliferative phenotype [43]. 

### 2.7. ECM Stiffness and Cancer

#### 2.7.1. Cancer Models

**Figure 1:**
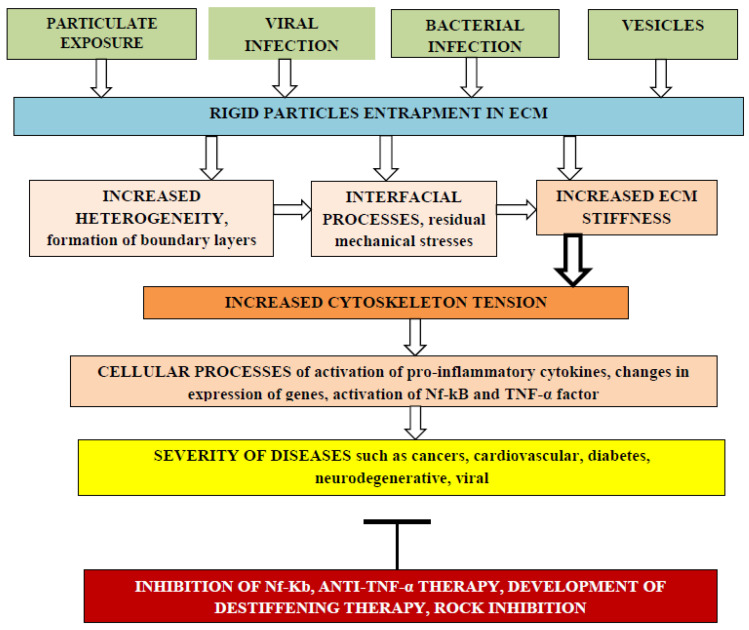
The effect of rigid particle entrapment by the ECM on the risk of adverse cellular processes that increase severity of various diseases. Different kinds of rigid particles such as nanoengineered particles, particles from air pollution, viruses, bacteria, and extracellular vesicles can be entrapped by the ECM, changing matrix heterogeneity due to the formation of interfacial regions with different elasticity moduli and thermal expansion coefficients. Internal residual tensile and compressive mechanical stresses can arise in the ECM, leading to enhanced stiffness that can be transmitted into cells through integrins, increasing cytoskeleton tension with activation of pro-inflammatory cytokines, changes in expression of genes, activation of the Nf-kB pathway, and expression of TNF-α and IL-6 pro-inflammatory factors. Such cellular processes increase the severity of various diseases and stimulate the development of adverse process inhibition methods.

Cellular biochemical processes depend on ECM mechanical stiffness. Entrapment of rigid particles can enhance ECM stiffness due to interfacial changes near rigid surfaces and cause various diseases including initiation and progress of different cancers, as shown in Figure 1.

Cancer development and progression can be associated with remodeling of the ECM. The layer-by-layer coating terminated by hyaluronic acid on calcium carbonate nanoparticles can be applied for targeting breast cancer cells. Hyaluronic acid (HA) is known as a ligand of the tumor-associated receptor CD44 [44]. The coating slows down the release of the encapsulated model drug. The authors concluded that future studies should focus on understanding the role of different HA receptors in nanoparticles’ accumulation in the extracellular matrix.

Cells regulate the structure and performance of the ECM; for example, cells secrete biomolecules that diffuse into the ECM, creating temporally and spatially controlled gradients that play an important role in inflammation and cancer [45]. HA is a main component of the ECM. In breast cancer, accumulation of low-molecular-weight HA and formation of local gradients play an important role in cancer development. Cellular response to gradients of HA oligomers can improve the understanding of HA-associated carcinogenesis. Soluble low-molecular-weight HA promotes the directional migration of cells. HA is partially immobilized by interactions with ECM components. Hydrogel HA gradients have been developed to screen cell behavior. HA gradients were developed through a two-step procedure: (i) diffusional deposition of colloidal gold nanoparticles to obtain a gradient and (ii) immobilization of end-on-thiolated hyaluronan on this gradient. The HA gradient is concomitant with alterations in hydrogel mechanical properties, and it is not at present clear whether the cell response is triggered by HA-activated signaling pathways or mechanosensing. HA gradients formed via surface immobilization of end-on thiolated HA (4.8 kDa) and gold gradients formed via diffusional deposition of colloidal nanoparticles were reported [46].

Hydrogels with various stiffness have been designed, and their influence on the morphology of mesenchymal stem cells (MSCs) and their differentiation was studied [47]. An artificial ECM for tumorigenesis research applications was reviewed [48]. 

It was shown that the time-dependent stiffness of methacrylated hyaluronic acid hydrogels can be dynamically modulated from “normal” (<150 Pascals) to “malignant” (>3000 Pascals) during an investigation of cultured mammary epithelial cells (MECs) in order to mimic and understand breast cancer development [49]. It was found that MECs begin to lose epithelial characteristics and gain mesenchymal morphology upon matrix stiffening.

It was reported in 2022 that 3D-bioprinted GelMA-nanoclay hydrogels induce colorectal cancer stem cells through activating wnt/beta-catenin signaling [50] and in 2016 that extracellular matrix stiffness dictates Wnt expression through the integrin pathway [51] by researchers at Tsinghua University and Lanzhou University, China, jointly with researchers at the University of California at San Diego, USA. The joint analysis of both articles allows the conclusion that nanoclay particles increase ECM stiffness, resulting in wnt/beta-catenin signaling activation and inducing colorectal cancer stem cells. Therefore, ECM de-stiffening could be beneficial in fighting various cancers.

Researchers at the University of Illinois, Chicago, USA, investigated the culture of epithelial ovarian cancer cells on three-dimensional collagen I gels in 2013 and reported that matrix rigidity activates Wnt signaling through down-regulation of the Wnt signaling inhibitor dickkopf-1 protein. The inverse relationship between dickkopf-1 and membrane type 1 matrix metalloproteinase was observed in human epithelial ovarian cancer specimens [52]. 

The inhibition of the Wnt/beta-catenin pathway in colon cancer cells by the most active vitamin D metabolite, 1alpha,25-dihydroxyvitamin D3 (1,25(OH)2D3), was reported. It was suggested that 1,25(OH)2D3 distinctly regulates two genes encoding the extracellular Wnt inhibitors DICKKOPF-1 (DKK-1) and DICKKOPF-4 (DKK-4). 1,25(OH)2D3 increases the expression of DKK-1, which acts as a tumor suppressor in human colon cancer cells and represses DKK-4 transcription, which is up-regulated in colorectal tumors, increasing cell migration and invasion [53,54].

Wnt signaling in brain tumors was analyzed in a recent review [55].

Three-dimensional hydrogel breast cancer models for studying the effects of hypoxia on the epithelial-to-mesenchymal transition were described. Hypoxia enhanced breast cancer cell migration and the expression of lysyl oxidase (LOX), which drives the crosslinking of collagen and elastin [56].

The advantages of 3D hydrogel cancer models and drawbacks of 2D cancer models were described [56]. In 2D models, cells adhere to a flat surface with only a part of the cells’ surface in direct contact with the substrate and with a lack of intercellular contacts. Compared with the cells localized in the 3D environment, the cells grown on the 2D stiff surface demonstrated different responses to biophysical cues, cytokine secretion capacity, and cell response to anti-cancer drugs [57,58]. It was demonstrated that MCF-7 cancer cells cultured in the 3D model had reduced sensitivity to doxorubicin in comparison with cells cultured in the 2D conditions [58]. The IL-8 expression was increased in human oral squamous cell carcinoma in the 3D environments but not in the 2D monolayers [59].

Increased ECM stiffness promotes nuclear localization of YAP and TAZ and upregulation of their target genes [60]. YAP/TAZ is essential for cancer initiation or growth [61].

#### 2.7.2. Polyphenols Decrease ECM Stiffness

The effect of polyphenols on the microbiome has been reviewed [62]. Polyphenols can inhibit NF-κB and TNF-α, which lead to the increase in tissue stiffness and permeability [63]. At least one aromatic ring with a hydroxyl group attached to it was mentioned as a common feature for polyphenols [64]. The polymers with such chemical structure can also be recommended for use as matrix plasticizers.

The beneficial health effect of polyphenols can be explained by their potential de-stiffening of the ECM due to the plasticizing effect that has been earlier demonstrated on edible polymer films [65]. The essential decrease in Young’s modulus was reported when gallic acid, p-hydroxy benzoic acid, and ferulic acid were used as plasticizers, but the lack of any considerable plasticizing effect for flavon was observed. 

The antiplasticizing effect of tannic acid incorporated into films from sorghum kafirin was observed with reduced elongation but increased tensile strength and Young’s modulus. The antiplasticizing effect could be due to the increased number of hydroxyl groups tightly binding within the matrix [66]. 

Films containing phenolic compounds also have antioxidant and antimicrobial properties.

Curcumin upregulates Nrf2 nuclear translocation [67,68]. The activation of Nrf2 by curcumin can inhibit the activation of tissue stiffness, inflammatory cytokine expression, and the NF-kB pathway [69,70]. 

Dietary polyphenols can decrease arterial stiffness [62], and it was reported that curcumin downregulates the pro-inflammatory cytokines TNF-α and IL-6 [71]. Thus, the joint consideration of these two articles allows arriving at the conclusion that ECM de-stiffening downregulates the expression of pro-inflammatory cytokines, and it has recently been reported that matrix stiffness exacerbates the pro-inflammatory responses of vascular smooth muscle cells [72]. 

Resveratrol and quercetin inhibit the NF-kB pathway [64]. The beneficial role of Nrf2 in cancer treatment has been reported [69,73,74,75].

It has been reported that Nrf2 downregulation contributes to epithelial-to-mesenchymal transition in *Helicobacter pylori*-infected cells [76]. The authors believe that the obtained “results could pave the way for new therapeutic strategies using Nrf2 modulators to reduce gastric carcinogenesis associated with *H. pylori* infection”. *Helicobacter pylori* infection is associated with oxidative stress. It is important to take into consideration that infection leads to an epithelial-to-mesenchymal transition (EMT), resulting in gastric carcinogenesis. 

**Figure 2:**
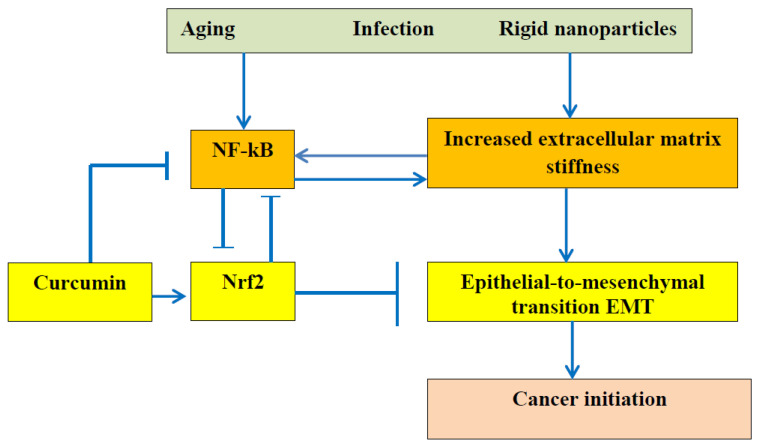
It is commonly accepted that dysregulation of NF-κB can lead to autoimmune diseases, chronic inflammation, and cancer. This scheme demonstrates the especially important role of ECM stiffness. Advanced age, bacterial and viral infection, and particles from air pollution reinforce the ECM, and increased stiffness activates the NF-kB pathway and causes epithelial-to-mesenchymal transition, leading to cancer initiation. The polyphenol curcumin can inhibit NF-κB due to Nrf2 activation and can retard cancer initiation.

The relationship between the ECM stiffness and EMT is presented in Figure 2.

It can be suggested that any infection particles entrapped in the ECM could initiate EMT and cancer as a result of changes in ECM stiffness. Commonly, the decrease in Nrf2 with increased oxidative stress can be associated with activation of the NF-kB pathway with enhanced inflammation [38].

It was found that infected tissues are softer than uninfected ones, and this was explained at least partially by cell cytoskeleton remodeling [77]. Researchers from the Medical University of Bialystok consider that the “molecular mechanism of gastric cancer development related to *Helicobacter pylori (H. pylori)* infection has not been fully understood, and further studies are still needed”.

#### 2.7.3. Chitosan and Chitosan Oligosaccharides

The use of injectable in situ chitosan hydrogels in cancer treatment was reviewed [78]. It is generally believed that cancers may result from the interaction between healthy cells and carcinogens such as ultraviolet (UV) radiation, chemical carcinogens such as asbestos and tobacco smoke, and biological carcinogens such as infections by viruses and contamination of food by mycotoxins. The authors considered that external agents cause genetic alterations resulting in mutations and thereby perturbing normal cell function. But the effect of the above-mentioned agents on the ECM must also be taken into consideration. 

Chitosan-based hydrogels possess the required good biocompatibility, mucosal adhesion, and hemostatic activity with potential for application in tissue engineering and drug delivery [79]. The fact that extracellular pH values in tumors are lower than in healthy tissues (pH = 7.4) [80] can be used in drug delivery systems. An injectable chitosan-chondroitin sulfate hydrogel with embedded kartogenin-loaded microspheres as an ultrasound-triggered drug delivery system for cartilage tissue engineering was designed and fabricated. It was observed that the hydrogel/microspheres’ compressive elastic modulus was greatly enhanced, which, in the authors’ opinion, is good for cartilage healing [81], though it would be beneficial to take into consideration the potential increased risks of inflammation and oxidative stress. A stiff ECM increases intracellular reactive oxygen species and activates oxidative stress.

#### 2.7.4. Anti-TNF-α and Anti-NF-κB Therapies 

Anti-TNF-α therapy decreases ECM stiffness and can be used in the treatment of age-related diseases such as rheumatoid arthritis [38,63,82]. The nuclear factor-κB (NF-κB) signaling pathway is involved in inflammation through the regulation of cytokines’, chemokines’, and adhesion molecules’ expression. A number of anti-inflammatory and anti-rheumatic drugs, such as glucocorticoids, aspirin, sodium salicylate, sulfasalazine, and gold compounds, have been mentioned as inhibitors of NF-κB activation [83]. Nutrients such as Omega 3 and Vitamin D can decrease NF-kB activation [84].

### 2.8. Equivalence of ECM Stiffness, Oxidative Stress, and Inflammation

The NF-kB pathway is identified as one of the main inflammatory pathways.

Human dermal fibroblasts seeded on a soft matrix demonstrated high oxidative stress resistance, due to high expression of Nrf2 [85]. 

**Figure 3:**
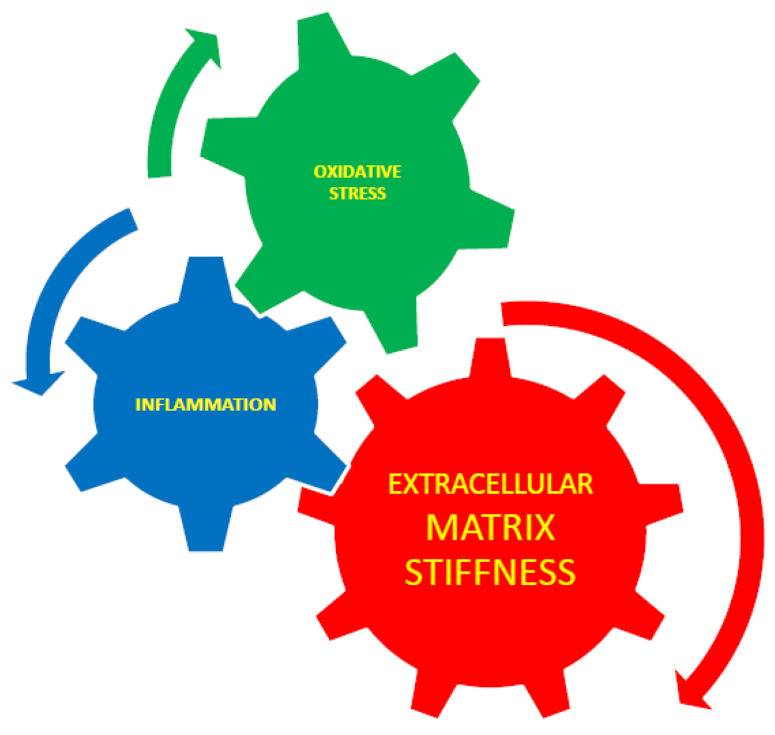
The interaction between extracellular matrix stiffness, inflammation, and oxidative stress. Extracellular matrix stiffness plays an essential role in the control of inflammation and cytoskeletal rearrangement, and it is known that inflammation causes stiffness [63] through TNF-α-dependent vascular inflammation. ECM stiffness is interrelated with oxidative stress. Inflammation and oxidative stress are also interrelated [38].

It has been reported that oxidative stress initiates arterial stiffness [86] and that stiffness can initiate oxidative stress [87]. Therefore, it can be suggested that oxidative stress and tissue stiffening are not two separate processes but two different aspects of a united process measured using different methods. The interaction between extracellular matrix stiffness, inflammation, and oxidative stress is presented schematically in Figure 3. As a third aspect, inflammation can be added to this united process because it was suggested that oxidative stress and arterial stiffness jointly lead to the development of diabetes.

Angiotensin-converting enzyme inhibitors (ACEIs) decrease arterial stiffness [88]. The role of the renin–angiotensin–aldosterone system (RAAS) in the inhibition of arterial stiffness was discussed. The excessive stimulation of angiotensin type 1 (AT1) receptors leads to oxidative stress and vascular inflammation. RAAS inhibition decreases arterial stiffness [89]. The effects of two RAAS inhibitors (quinapril and aliskiren) and two beta-blockers (atenolol and nebivolol) on arterial stiffness, measured using pulse wave velocity (PWV), have been reported [90]. The PWV was decreased by quinapril, aliskiren, nebitovol, and atenolol after 2 weeks, and continued to decrease till 10 weeks in patients on quinapril, aliskiren, and nebivolol but did not change in patients taking atenolol. Angiotensin receptor blockades (ARBs) reduce arterial stiffness as measured using pulse wave velocity (PWV) [91].

## 3. Conclusions and Future Perspective 

The reported results have potential to initiate investigation in emerging directions with the aim to develop effective therapies in diseases dependent on ECM stiffness using hydrogels as ECM mimics.

Rigid particles incorporated into the ECM can represent health risks. The accumulation of rigid particles, such as nanofillers for polymer composites, particulate matter from air pollution, extracellular vesicles, exosomes, viruses, bacteria, and other entrapped particles, increases ECM stiffness, leading to the initiation of related diseases. 

The function and effect of particulate and fiber fillers in particle-reinforced hydrogel composites, fiber-reinforced hydrogel composites, and anisotropic filler-reinforced hydrogel composites can be investigated in more detail in the near future, paying special attention to the risk imposed by the effect of mechanical reinforcement on cellular processes at the molecular level. The risks related to increased ECM stiffness as a result of rigid nanoparticle incorporation must be studied in more detail. Nanoclay particles increase ECM stiffness, resulting in wnt/beta-catenin signaling activation, and can induce colorectal cancer stem cells. Wnt signaling-targeting drugs are still not clinically available. Additional efforts will be needed to demonstrate the real clinical impact of Wnt inhibition in various tumors.

Vitamins that possess an antioxidant function, such as vitamins A, C, and E, and polyphenols are also expected to decrease ECM stiffness in the treatment of ECM-dependent diseases. The mechanism of vitamins’ beneficial effect must be suggested. The heterogeneous structure of the ECM leads to the development of internal residual stresses in the ECM. The relationship between external mechanical stresses and internal cellular stresses and mechanism of mechanical stress transmission from the ECM to cells is still elusive. The contribution of internal residual stresses to mechanotransduction has to be confirmed.

The role of various water populations in normal and malignant tissue must be further investigated using modern physical methods, such as magnetic resonance. The essential advancement from mechanobiology to mechanomedicine can be expected in the future. The links, correlations, and interaction between inflammation, oxidative stress, and tissue stiffness must be further investigated in more detail.

## Data Availability

Not applicable.

## Abbreviations

ACEIs	angiotensin-converting enzyme inhibitors
ARBs	angiotensin receptor blockades
AT1	angiotensin type 1
CS-PEO	chitosan-polyethylene oxide
ECM	extracellular matrix
FACs	focal adhesion complexes
GelMA	gelatin methacrylate
GNR	gold nanorod
HA	hyaluronate
IL-6	interleukin 6
LOX	lysyl oxidase
MECs	mammary epithelial cells
MMPs	matrix metalloproteinases
MSCs	mesenchymal stem cells
NC-ALG	nanoclay–alginate
NF-kB	nuclear factor kappa light-chain enhancer of activated B cells
Nrf2	the nuclear factor erythroid 2-related factor 2
PAA	poly(acrylic acid)
PEG	poly (ethylene glycol)
PVA	poly(vinyl alcohol)
PWV	pulse wave velocity
QENS	quasi-elastic neutron scattering
RAAS	renin–angiotensin–aldosterone system
TIMPs	tissue inhibitors of matrix proteases
TNF-α	tumor necrosis factor α
UV	ultraviolet
Wnt	wingless-related integration site
